# Innovations in gene and growth factor delivery systems for diabetic wound healing

**DOI:** 10.1002/term.2443

**Published:** 2017-09-21

**Authors:** Ashang Luwang Laiva, Fergal J. O'Brien, Michael B. Keogh

**Affiliations:** ^1^ Tissue Engineering Research Group, Department of Anatomy Royal College of Surgeons in Ireland Dublin Ireland; ^2^ Medical University of Bahrain Adliya Kingdom of Bahrain; ^3^ Trinity Centre for Bioengineering Trinity Biomedical Sciences Institute, Trinity College Dublin Ireland; ^4^ Advanced Materials and Bioengineering Research Centre Royal College of Surgeons in Ireland and Trinity College Dublin Ireland

**Keywords:** biomaterials, diabetic foot ulcer, gene, growth factors, wound healing

## Abstract

The rise in lower extremity amputations due to nonhealing of foot ulcers in diabetic patients calls for rapid improvement in effective treatment regimens. Administration of growth factors (GFs) are thought to offer an off‐the‐shelf treatment; however, the dose‐ and time‐dependent efficacy of the GFs together with the hostile environment of diabetic wound beds impose a major hindrance in the selection of an ideal route for GF delivery. As an alternative, the delivery of therapeutic genes using viral and nonviral vectors, capable of transiently expressing the genes until the recovery of the wounded tissue offers promise. The development of implantable biomaterial dressings capable of modulating the release of either single or combinatorial GFs/genes may offer solutions to this overgrowing problem. This article reviews the state of the art on gene and protein delivery and the strategic optimization of clinically adopted delivery strategies for the healing of diabetic wounds.

## 1 INTRODUCTION

Diabetes has become an ever‐escalating global health crisis. Rapid urbanization, transition in nutritional status and increasing sedentary lifestyles are often mentioned as the cause for dynamic rise in the epidemic (Hu, 2011). In 2014, 387 million people were estimated to suffer from diabetes and it accounted for 11% of global health expenditure or at least $612 billion (da Rocha Fernandes et al., 2016). A study reported recently in *Diabetes Research and Clinical Practice*, estimated that by 2035, the number of diabetes‐affected people would reach at least 592 million people worldwide, with two‐thirds of all diabetes cases occurring in low‐ to middle‐income countries (Guariguata et al., 2014). Given such statistics and with no curative solution, diabetes poses serious threat to the economies of both developed and developing nations.

The expenses involved for the treatment of multiorgan dysfunction resulting from vascular (macro and micro) complications in diabetic patients could be held responsible for the huge burden imposed upon the global economy (American Diabetes Association, 2013). Chronic hyperglycaemia is known to be the main factor for the initiation of diabetes‐associated vascular complications. Under such pathological conditions, an injury to the skin could cause serious life‐threatening risk due to the loss of innate healing mechanism of the skin. Diabetic patients with foot wounds often fall victim to such risk. Histopathological features of wounds in diabetic foot are identified by abnormal microvessels that can be cuffed with collagen, laminin, fibronectin or fibrin in the wound edges (Mendoza‐Mari et al., 2013). Further accumulation of debris can exacerbate the development of ulceration due to increased pressure at the wound edges, a feature predominantly observed in diabetic neuropathy, where an individual loses protective pedal sensation (Krishnan, Quattrini, Jeziorska, Malik, & Rayman, 2007). This population is prone to develop chronic nonhealing diabetic foot ulcers (DFUs), which are estimated to occur in 15% of all persons with diabetes. DFUs precede 85% of all diabetes‐related lower extremity amputations and presents as a significant mortality risk factor (Hoffstad, Mitra, Walsh, & Margolis, 2015; Snyder & Hanft, 2009). Timely prevention and healing of diabetic ulcerations form the baseline for amputation prevention and reduction in mortality rate. Currently, debridement in conjunction with infection control, off‐loading to relieve pressure, and maintenance of a moist wound bed has been the standard wound‐care practice for DFUs. Continual deployment of the multidisciplinary wound‐care setting is considered favourable for healing by converting chronic wounds into acute, but the consistently poor vascular status necessitates longer hospitalization stays, incurring high expenditure owing to incremental resource use, including hospitalization charges (Driver, Fabbi, Lavery, & Gibbons, 2010). Despite high initial costs, revascularization using advanced treatment modalities such as growth factors (GFs), hyperbaric oxygen therapy and bioengineered skin grafts have gained a faster pace than conventional wound care in achieving faster wound closure and reducing ulcer recurrence, eventually improving the overall quality of life of DFU patients (Snyder & Hanft, 2009). However, the optimal treatment regimen is subject of much debate. One reason for this is that the involvement of diverse mechanisms in impairing physiological regulations in the diabetic wound bed presents a barrier in elucidating the effective route for treatment. Clearly, however, considering the urgent treatment need, therapies targeted to improving the angiogenic pathways have significant potential in stimulating vascularization and accelerating healing. Here, we aim to provide insight into *in vivo* delivery of biomolecular therapeutics, particularly GFs and discuss their efficacy in activating the angiogenic pathways for timely healing of wounds in diabetic patients.

### 1.1 Impaired angiogenesis in diabetic wounds

**Figure 1:**
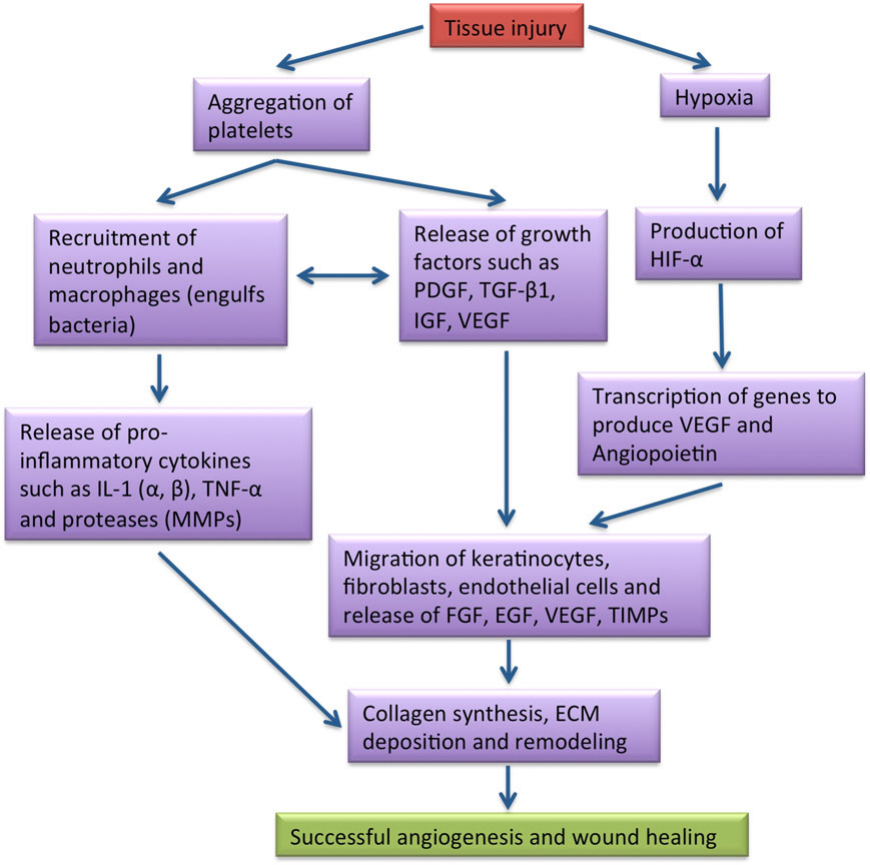
A schematic depicting normal wound healing process. TIMPs = tissue inhibitors of metalloproteinases

Angiogenesis is characterised by the sprouting of new blood vessels from pre‐existing vessels and is a critical step in wound healing as it allows provision of oxygen and nutrients via the blood streams to inhibit apoptosis of vital cells in the injured tissue. Immediately, in response to tissue injury, a typical angiogenesis follows a complex multistep process involving extracellular matrix (ECM) degradation, proliferation, survival, migration and morphological changes of endothelial cells (ECs) and their anastomosis to assemble into a mature vasculature, which encompasses concurrently with overlapping wound healing phases of coagulation, homeostasis, inflammation and proliferation with matrix deposition and remodelling (Li, Zhang, & Kirsner, 2003; Velnar, Bailey, & Smrkolj, 2009). A schematic of a typical wound healing process is presented in Figure 1. In chronic wounds, the progressive synchrony of the healing phases is lost and the process of angiogenesis remains stalled in the inflammation or proliferation phase leading to the formation of impaired granulation tissue (Falanga, 2005). Briefly, angiogenesis in its inflammation phase commences with the production of nitric oxide (NO) by inflammatory cells (e.g. macrophages), which in turn stimulates vasodilation and permeability, facilitating immune cells (e.g. neutrophils) extravasation and release of proinflammatory cytokines and chemokines, which further activates neighbouring ECs and fibroblasts to release angiogenic factors. The inflammatory cells themselves would release angiogenic factors, such as vascular endothelial GF (VEGF), fibroblast GF (FGF), hepatocyte GF, epidermal GF (EGF), transforming GF‐β (TGF‐β) and angiopoetin, and cytokines such as tumour necrosis factor‐α, which can stimulate angiogenesis following accumulation at the site of inflammation. Elevation in hypoxia is another feature in inflammation which is characterized by the production of hypoxia inducing factors (HIFs), which further promotes transcription of angiogenic genes such as VEGF and angiopoetin‐2 (Costa, Incio, & Soares, 2007; Polverini, 2012). However, in hyperglycaemia, these mechanistic events of inflammation‐mediated angiogenesis are interfered upon by accelerated accumulation of advanced glycation end products (AGEs), whose formation as a result of nonenzymatic glycation of proteins, lipids and nucleic acids may occur both extra‐ or intracellularly (Singh, Bali, Singh, & Jaggi, 2014).

**Figure 2:**
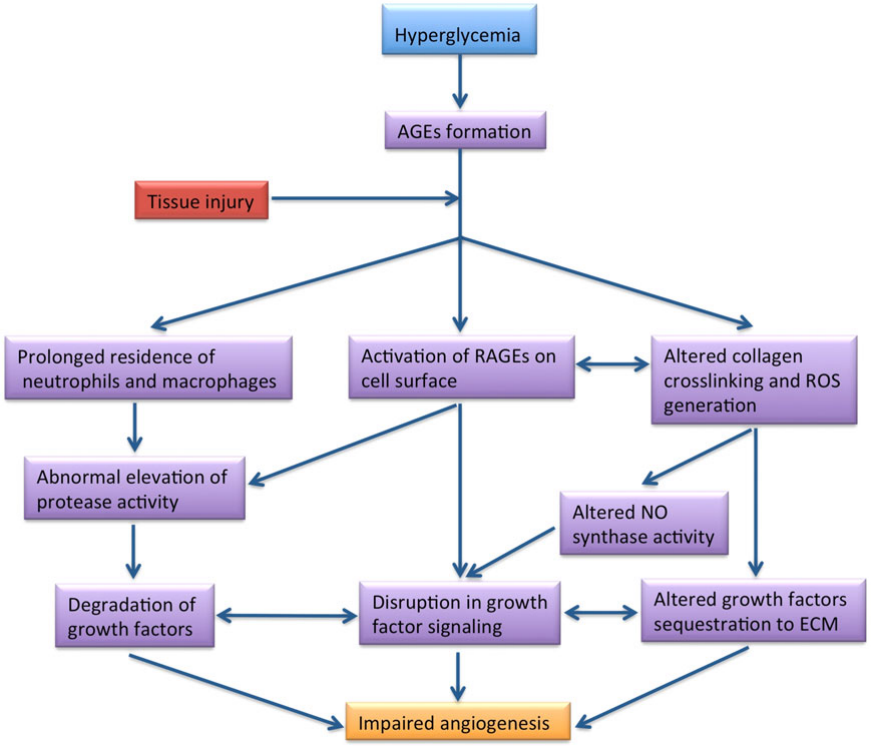
Hyperglycaemia impairs angiogenesis and wound healing

Within the microvasculature, AGEs cause aberrant crosslinking of the ECM proteins (such as collagen) altering their binding affinity for proteoglycans and sensitivity to collagenase. This event in turn, drives the generation of reactive oxygen species, which interferes with the production of NO by endothelial NO synthase. By contrast, AGEs induce the expression of their receptors on the surface of various cell types, whose interaction with the circulating AGEs activates a myriad of abnormal intracellular signals (mainly the nuclear factor‐κB pathway), perpetuating proinflammatory and matrix metalloproteinase activities, and elevation of oxidative stress, further contributing to AGEs deposition (Pierce, 2001; Stirban, Gawlowski, & Roden, 2014). While this pathological loop is at play, GF sequestration to the ECM is altered and the abnormal elevation in proteolytic activity degrades the GFs substantially, resulting in local depletion of GFs, inhibiting angiogenesis and healing (Figure 2) (Briquez, Hubbell, & Martino, 2015). Furthermore, accumulation of AGEs is believed to cause segmental demyelination in the nerves leading to interruption in axonal transport and alter the expression of neuronal peptides such as nerve growth factor (Apfel et al., 1998). The resulting dysfunction of sensory nerve fibres is known to compromise the immunomodulation of the skin during the inflammatory phase of wound healing (Pradhan, Nabzdyk, Andersen, LoGerfo, & Veves, 2009). Hyperglycaemia also has an adverse effect on the degree of erythrocyte aggregation and deformability, and haemorheology thereby impeding blood flow to the distal ends of the wound, limiting the supply of oxygen (prolonging hypoxia) and nutrients for tissue homeostasis (Cho, Mooney, & Cho, 2008). Collectively, these events imply a portal for supplementation of exogenous GFs, which in turn would stabilize angiogenesis and induce normal healing in diabetic wounds.

## 2 GROWTH FACTOR‐THERAPY FOR DIABETIC WOUND HEALING

Applications of exogenous GFs are considered a promising approach for the treatment of most chronic or degenerative disorders. The rationale for using GFs has been attributed to its ability to trigger and coordinate a myriad of cellular and molecular events, which is critical for successful healing of injured tissues (Barrientos, Stojadinovic, Golinko, Brem, & Tomic‐Canic, 2008). A range of GFs including platelet derived GF (PDGF), VEGF, EGF, FGF, TGF, keratinocyte GF (KGF), insulin‐like GF and HIF have been well documented in reviews for their potential in accelerating the wound healing process (Bennett, Griffiths, Schor, Leese, & Schor, 2003). Of these, PDGF, VEGF, EGF, FGF and TGF‐β1 have been applied in clinical trials for the treatment of DFU (Martí‐Carvajal et al., 2015).

Clearly, nerve fibre loss in diabetic neuropathic patients is a well‐known factor that contributes to impaired healing of diabetic wounds, but the understanding on the influence of neuropathic condition in impaired healing still remains warranted. Nevertheless, topical application of nerve growth factor formulations into diabetic wounds have been demonstrated to promote regeneration of nerves and induction of reparative angiogenesis mediated by recruitment of ECs to the wound site (Graiani et al., 2004; Muangman et al., 2004). Moreover, Thomson et al. (2010), using a nonhuman primate model, demonstrated for the first time that connective tissue growth factor (CTGF) was also dysregulated in diabetic wounds. Later in 2015, it was reported that topical application of CTGF could promote healing through re‐epithelialization in diabetic rodents (Henshaw, Boughton, LoMcLennan, & Twigg, 2015).

### 2.1 Growth factor delivery routes adopted in clinical studies for DFU

Early clinical trials with GFs for DFU dates back to mid‐1990s with that of Becaplermin (Regranex®), a topical gel formulation of recombinant human (rh) PDGF‐BB. It is the first GF approved by the US Food and Drug Administration for use in DFUs. However, its progression to actual clinical practice remains unmet, as reported efficacies were based on small randomized trials which were performed under well controlled environments, failing to yield evidence‐based guidelines specifying the time and required mode of treatment. Furthermore, the difficulty with routine clinical experience and less successful outcomes have prompted the search for an alternative mode of delivery that can prolong the exposure of Becaplermin alone or in combination with other GFs to the wound site (Papanas & Maltezos, 2008). Additionally, applicability of topical gel formulation becomes limited with increasing severity of ulcers as the GFs undergo proteolytic breakdown leading to inadequate diffusion to the deeper wound layers. With the development of topical spray form, the above limitation appears to have been compensated to a certain extent, as it allows early maximal contact of the GFs with the entire wound surface. The therapeutic efficacy was demonstrated in a clinical study where topical spraying of rhEGF (Easyef®) twice daily resulted in wound size reduction over 80% by the 8^th^ week irrespective of the grade of ulcer (Tuyet et al., 2009). Alternatively, intralesional injection offers a relatively painful but more localized route for GF delivery. Acosta et al. (2006), performed the proof‐of‐concept trial and showed that intralesional injection of rhEGF into DFUs of Wagner's Grade 3 or 4 (ulcer area > 20 cm^2^) thrice weekly, received appreciable granulation response and wound closure rate with enhanced angiogenesis. Their study was further validated in multicentre and placebo‐controlled trials (Fernández‐Montequín et al., 2007; Fernández‐Montequín et al., 2009). Taken together, these clinical studies are representative of the feasibility of an easy *in vivo* approach (injection or topical) for therapeutic delivery to diabetic wounds. However, serious concern exists that might interfere with the benefit–risk balance of the treatment regime, and halt the progression from bench to bedside. For example, there is dose‐dependent (more than three tubes) risk of cancer stimulation with Becaplermin application (Papanas & Maltezos, 2010). By contrast, topical spraying or intralesional injection requires frequent disruption of the dressing, that is essentially used to occlude the wound from transference of microorganisms from other external environmental sources (Mertz, Marshall, & Eaglstein, 1985). Although the level of risk of infection with the above practice is unclear, termination of treatment resulting from infection have been reported (Fernández‐Montequín et al., 2007; Tuyet et al., 2009).

Achieving favourable benefit–risk balance remains the key to clinical translation. The need to overcome the limitations described above has led to exploring into novel GF delivery systems/techniques that serve to protect the GFs from degradation but at the same time allow controllable release and reduce the frequency of administration (Gainza, Villullas, Pedraz, Hernandez, & Igartua, 2015). Alternatively, gene delivery approaches that use deoxyribonucleic acid (DNA) encoding for therapeutic genes could potentially provide a more stable and effective approach to allow sustained and controlled release of therapeutic factors (O'Brien, 2011). The following sections will discuss the various approaches adopted for the delivery of GFs and genes, as applied to diabetic wound healing.

## 3 BIOMATERIAL SYSTEMS AS DEPOTS FOR THERAPEUTICS DELIVERY TO DIABETIC WOUNDS

The growing interest in the development of biomaterial systems for therapeutic delivery to diabetic wounds can be attributed to their ability to sequester and release clinically significant doses of the therapeutics for an extended period of time within the targeted site. Some of the existing challenges with designing biomaterials for such applications include the maintenance of GF structure and bioactivity during fabrication, high encapsulation efficiency, bioavailability and achieving complete release with a therapeutically active pharmacokinetic profile from the biomaterial depot. Understanding the time‐ and dose‐dependent response to individual GFs is also important. For example, it is acknowledged that VEGF‐A requires a higher initial release for initiation of angiogenesis, followed by steady but lower release rate maintained within the therapeutic window, while EGF requires prolonged exposure to be effective (Amsden, 2015). In line with this, the feasibility of designing a novel biomaterial depot with the ability to tailor the release kinetics of two distinct GFs, corresponding to their effects on stabilizing angiogenesis was well demonstrated as far back as 2001. This study also stands as a notable example that bolus delivery of multiple GFs is not sufficient to sustain angiogenesis (Richardson, Peters, Ennett, & Mooney, 2001). Additionally, the fact that GFs have short half‐life, limited diffusion lengths and very low concentration (10^–9^ to 10^–11^ m)‐associated bioactivity necessitates the persistent presence of the biomaterial depot within or in the implanted site for extended time frames (from days to weeks) without inducing host‐immune response. This condition primarily requires that the biomaterials be highly biocompatible however, biomaterials that can be degraded and excreted or resorbed into host tissues are gaining utmost importance for the development of implantable systems.

The use of degradable biomaterials eliminates the need for a second surgical intervention for implant removal, but also allows for improved healing by facilitating tissue ingrowth into the degrading construct. As the degradation is believed to be mediated by specific biological activity, the process is generally termed biodegradation. Currently, the commonly used biodegradable biomaterials for therapeutic applications include synthetic polymers of polyester family such as polyglycolic acid (PGA), polylactic acid, poly(lactic‐co‐glycolic acid) (PLGA), polycaprolactone (PCL) and/or polymers of natural origin, namely collagen, gelatine, fibrin, hyaluronic acid, dextran, alginate, and chitosan. The synthetic polymers are known to biodegrade mainly via cleavage of hydrolytically sensitive ester bonds in the polymer while that of natural polymers are often enzymatic. The erosion behaviour effects therapeutic release kinetics in a biomaterial making biodegradability a fundamental part of biomaterial implants (Amsden, 2015; Nair & Laurencin, 2007). However, due to the variations in site‐to‐site and pathological conditions in patients, the biomaterial system may need to be uniquely tailored for controlled degradation *in situ* (Nair & Laurencin, 2007). Developing a biomaterial‐based delivery systems with predictable degradation kinetics will enable one to control the release profile of therapeutics, resulting in localization of optimized concentrations of therapeutics (Lee, Silva, & Mooney, 2011). Typically, the degradation kinetics of the biomaterial systems are determined *in vitro* by incubating it in either phosphate‐buffered saline or simulated body fluid solutions. However, in many cases, the results of *in vitro* have not been reflected *in vivo* (Bölgen, Menceloğlu, Acatay, Vargel, & Pişkin, 2005; Lu et al., 2000). Degradation studies conducted in an environment that closely mimics the *in vivo* environment would give valuable insights for the development of more precisely controllable biomaterial‐based delivery systems. For instance, knowledge on the physicochemical properties of the site of implantation and its duration of contact with the tissues and body fluids could help choose the optimum incubation media and the duration of study (Azevedo & Reis, 2005). Keeping note of the above highlights, herein we discuss the different forms of therapeutic biomaterial systems and its efficacy in the treatment of diabetic wounds.

### 3.1 Particulate systems for sustained release of GFs into diabetic wounds

Biodegradable polymeric particles are one of the most explored delivery systems for site‐specific controlled release of therapeutics. The emulsion/solvent extraction methods are the most commonly employed to prepare polymeric particles of various sizes [with the range of 1–1000 μm for microparticles and <1 μm for nanoparticles (Zhang & Uludağ, 2009)]. Due to the high solubility of GFs in water, the emulsification process is designed to yield a polymeric system with GFs embedded in the hydrophilic phase while the hydrophobic phase assembles as the protective shell/layer. Typically, the GFs release curve from these systems exhibits a biphasic pattern with an initial burst release followed by a gradual and sustained release. However, an improved control over the release rate is deemed achievable by developing systems with predefined diameter or shell thickness. Often, smaller particles are known to exhibit faster initial release of therapeutics than larger particles. This behaviour by smaller particles is thought to result from a combined effect of early diffusion of therapeutics owing to the shorter penetration length of water to the centre of particle and higher surface‐area to volume ratio facilitating increased efflux of therapeutics (Kim & Pack, 2006). Clearly, optimization of the fabrication parameters to yield reproducible particles of desired size will contribute significantly in the field of polymeric particles‐based delivery systems.

A double‐emulsion method was employed to develop rhEGF loaded PLGA nanoparticles. The particles exhibited an encapsulation efficiency of 85.6% but a short release period lasting only up to 24 hours *in vitro*. The rhEGF‐PLGA nanoparticles were sprayed once daily into the wound of diabetic mice but failed to induce any healing‐response until the 3^rd^ day of treatment. However, within 7–21 days of study, the group treated with rhEGF‐PLGA nanoparticles exhibited the fastest healing rate as compared to groups treated with rhEGF or PLGA alone. The accelerated healing was attributed to the maintenance of an effective local concentration of bioactive rhEGF released from the PLGA nanoparticles (Chu et al., 2010). A study by Chereddy et al. (2015) adopted the intradermal route for the delivery of VEGF‐loaded PLGA nanoparticles. *In vitro* study found that the PLGA nanoparticles could sustain the release of VEGF for a period of 30 days. The groups treated with VEGF‐loaded PLGA nanoparticles demonstrated the maximum healing response when compared to VEGF or PLGA alone (Chereddy et al., 2015). Minimizing the frequency of therapeutics administration is one of the important aspects of an effective therapy. Gainza, Aguirre, Pedraz, Hernández, and Igartua (2013) reported that the use of PLGA‐alginate microspheres as GFs carriers could significantly minimize the frequency of administration. In their study, single intralesional injection of PLGA‐alginate microspheres carrying a dose of 75 μg rhEGF was found to promote complete re‐epithelialization in diabetic rats by 11 days (Gainza et al., 2013). Lipid based‐particulate systems have also been employed for the delivery of GFs via topical or injection routes. Because of their resemblance with the biological membranes, lipids are particularly attractive for topical application. Cellular internalization of the lipid nanocarriers (solid lipid nanoparticles‐SLNs or nanostructured lipid carriers‐NLCs) has been demonstrated *in vitro*. The significance of this feature was evident on day 8 post‐treatment in diabetic mice, where application of two low topical dosage forms of rhEGF loaded SLNs (10 or 20 μg) or NLCs (20 μg) exhibited greater re‐epithelialization than groups treated with two intralesional dosage of free 75 μg rhEGF (Gainza et al., 2014).

**Figure 3:**
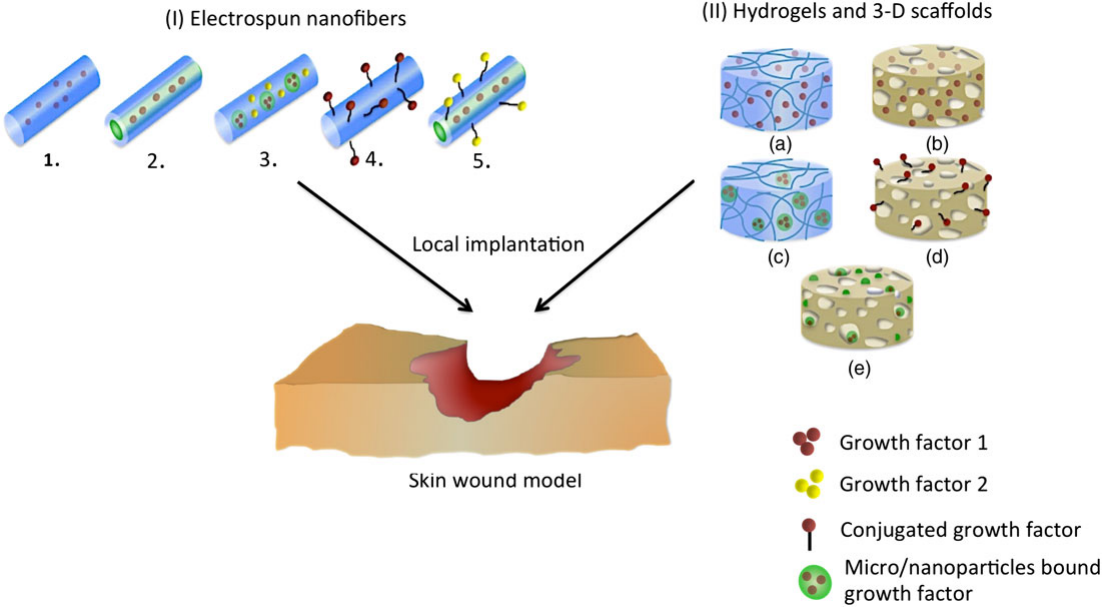
The different formats of commonly adopted therapeutic dressings. (I) Electrospun nanofibres 1. Direct blending of GFs 2. Encapsulation of GFs in the core of a core‐shell construct 3. Incorporation of nanoparticles bound GFs 4. GFs conjugated on the surface of fibres 5. Encapsulation of GFs in the core of a core‐shell followed by surface‐conjugation of another GF. (II) Hydrogels and 3D scaffolds. (a) Entrapment of GFs within the hydrogel matrix; (b) entrapment of GFs within the porous scaffold; (c) micro/nanoparticles bound GFs embedded into hydrogel; (d) GFs chemically conjugated onto scaffolds; (e) incorporating micro/nanoparticles bound GFs into scaffold

The studies described above are representative of the various administrative routes applicable with particulate systems. Considering the relative noninvasiveness of the topical approach, identifying the feasibility of using particulate systems for topical GFs delivery as well as an occlusion dressing has become extremely relevant. With large contact surfaces and high bioadhesiveness, and good moisture permeation properties, the particulate systems are logically ideal for topical application. However, the need for frequent topical administration with the particulate systems (Chu et al., 2010) in the attempt to offer optimum therapeutic response may affect patient compliance, for instance, causing frequent disturbance to the wound from unnecessary dressing changes. Therefore, in line with this problem, development of locally implantable therapeutic dressings represents a potential solution. The availability of a wide range of biomaterials and the emergence of advanced fabrication tools and techniques serve as the backbone to finding this solution. The commonly used biomaterials dressings are shown in Figure 3. Some notable instances following the application of these dressings are discussed in the following sections. A summary of the corresponding wound healing outcome is also summarized in Table 2.

### 3.2 Modulating the release of GFs from polymeric fibrous mats

Therapeutic polymeric nanofibrous mats generated using electrospinning is a focus of investigation as wound dressings. These nanofibrous mats intrinsically possess high porosity and surface area which are essential for facilitating the permeation of oxygen and efficient absorption of fluids (Zahedi, Rezaeian, Ranaei‐Siadat, Jafari, & Supaphol, 2010). Typically, biomolecules are blended into a polymer solution, after which the mixed solution is spun to generate the nanofibre‐based delivery systems. Lee et al. (2015) used this simple approach to develop rhPDGF‐blended PLGA nanofibres and showed that a prolonged and sustained release of rhPDGF occurred for 21 days and significantly induced complete wound closure in diabetic rats. Preservation of GF bioactivity against the high proteolytic activity of wound bed is one of the greatest challenges in GF therapy. Choi, Leong, and Yoo (2008) suggested that surface‐conjugation of GFs onto the nanofibres is an effective strategy to preserve GF bioactivity following implantation *in vivo*. They found that implantation of rhEGF conjugated PCL or PCL‐PEG nanofibres promoted re‐epithelialization through the preservation of keratinocytic phenotype within the wound site. The latter effect was believed to result from strong binding of EGF receptors on keratinocytes with the EGF on nanofibres (Choi et al., 2008). The ability to generate core‐sheath fibrous structures using emulsion or co‐axial electrospinning has broadened the applicability of nanofibres for GFs delivery. The technique allows encapsulation of GFs within the core‐phase of the fibrous construct thereby offering protection of GFs from early proteolysis and also, minimization of the initial burst release. An initial burst release as low as 14.0 ± 2.2% for bFGF encapsulated in the core of an emulsion electrospun poly(ethylene glycol‐co‐lactide) (PELA) nanofibres has been reported. Synchronously to epithelialization, the fibres were also found to degrade over time (Yang et al., 2011). A co‐axially spun core‐sheath construct capable of releasing dual‐GFs with different release profiles has also been shown to be promising as therapeutic dressing when tested in diabetic mice (Choi, Choi, & Yoo, 2011). The construct consisted of bFGF in the core with covalently immobilized EGF on the sheath layer. Early diffusion of bFGF through the thin sheath layer (100–300 nm) followed by the release of immobilized EGF via erosion of the sheath matrix were thought to control the release rate (Choi et al., 2011). Tailored release of multiple GFs was reported to induce accelerated wound closure rate, higher collagen deposition and enhanced maturation of vessels than dual‐GFs (bFGF and EGF) *in vivo*. The multiple GFs delivery construct was developed by simultaneous electrospinning of two different precursor solutions, each prepared by dispersing either VEGF‐loaded gelatine nanoparticles into bFGF‐collagen solution or PDGF‐loaded gelatine nanoparticles into EGF‐hyaluronic acid solution. When tested *in vitro*, early and faster release was observed for nanofibres‐dispersed GFs (bFGF and EGF) while nanoparticles‐bound GFs (VEGF and PDGF) were released in a slow and sustained manner (Lai et al., 2014).

Quite contrary to electrospinning, a group recently reported the use of a layer‐by‐layer technique to form a hydrolytically degradable tetra‐layer architecture consisting of poly(β‐amino esters), poly(acrylic acid), VEGF and/or PDGF and heparan sulfate over a woven nylon mesh. Electrostatically stacking the GFs between the layers of different polymers allowed the delivery of multiple GFs with distinct release kinetics via surface‐based erosion, facilitating a complementary effect of the individual GFs on wound healing (Almquist, Castleberry, Sun, Lu, & Hammond, 2015).

### 3.3 Development of GF loaded three‐dimensional biomaterial dressings

To date, the terms *sponge* and *foam* have been used interchangeably to represent soft and porous shape‐conformable materials. Due to their high absorbency and permeability to moisture and oxygen, both are being used as standard wound dressings (Moura, Dias, Carvalho, E., & de Sousa, 2013). However, the overlapping choice of these dressing materials with those for tissue engineering applications seems to have covered the disparity between the two forms by mere substitution with the term *scaffold*, which generally represents a temporary platform for guided neotissue formation.

By virtue of its abundant occurrence in native ECM, collagen has been an attractive material for the development of biomimetic scaffolds. Collagen‐based scaffolds alone (Moura et al., 2014) and in combination with other natural polymers such as gelatine (Kanda et al., 2014), hyaluronic acid (Kondo, Niiyama, Yu, & Kuroyanagi, 2012) and chitosan (Wang et al., 2008), or synthetics such as PGA (Nagato, Umebayashi, Wako, Tabata, & Manabe, 2006) have been examined for their candidacy as GFs‐releasing wound dressings. The freeze‐drying technique has been conventionally used to develop such scaffolds (Kondo et al., 2012; Moura et al., 2014; Nagato et al., 2006), which are subjected to crosslinking via chemical (Moura et al., 2014), ultraviolet (UV)‐irradiation (Kondo et al., 2012) or thermal treatment (Nagato et al., 2006) to induce structural stability. From a therapeutic perspective, combining collagen with other polymers to generate a composite scaffold is particularly attractive because it can offer improved resistance to collagenase digestion and also sustain the release of GFs with a slower rate (Wang et al., 2008). Interestingly, one study found that the resistance to degradation of a collagen–gelatine composite scaffold increased correspondingly with higher bFGF loading. However, their *in vivo* application found that the scaffolds containing 14 μg/cm^2^ bFGF induced complete epithelialization and formation of significantly higher density of capillaries than 50 μg/cm^2^ groups by 2 weeks. The high dose (50 μg/cm^2^) bFGF delivery was presumed to inhibit keratinocyte proliferation (Kanda et al., 2014).

For local implantation to wounds, incorporating nanoparticles‐bound GFs into scaffold is a plausible strategy for two main reasons. First, it would protect the GFs against wound proteases during early course of implantation and, second, delay the initial release until induction of healing. It has been reported that, with respect to free‐GFs loaded scaffolds, application of a fibrin‐based scaffold containing VEGF‐ or bFGF‐loaded PLGA nanoparticles delayed the initial release as well as onset of healing, but demonstrated similar wound closure rate as that of the former by day 15. It is also worth noting that wounds treated with GF loaded scaffolds were found to contain reduced numbers of inflammatory cells as compared to scaffolds without GFs (Losi et al., 2013). Furthermore, maintaining controlled hydration of the wound is essential for stimulating the migration of epidermal cells and epithelialization, and preservation of GFs and cytokines for wound repair (Junker, Kamel, Caterson, & Eriksson, 2013). Hydrophilic polyurethane (PU) formed by copolymerizing with PEG has been an attractive dressing material for maintaining good moisture conditions in the wound bed. A group had shown that a PU dressing loaded with rhEGF could exhibit a water vapor transmission rate of nearly 3000 g/m^2^/day, which was believed to be desirable for preventing excessive dehydration of the wound. Additionally, the PU dressing was found to sustain the release of rhEGF for up to 7 days *in vitro*. Implantation of the rhEGF loaded PU dressing promoted re‐epithelialization and complete recovery of the wound by 21 days in diabetic rats (Pyun do, Choi, Yoon, Thambi, & Lee, 2015). Based on a previous report that collagen‐binding domain (CBD)‐linked VEGF exhibited high affinity for collagen and retainability at granulation tissue (Yan et al., 2010), Tan et al. (2014) reverse‐applied the concept to develop a collagen scaffold system capable of retaining exogenous VEGF from being washed away by the wound exudates. At 7 days postimplantation, CBD‐VEGF loaded collagen scaffold induced significantly higher density of blood vessel formation than native VEGF loaded scaffolds reflecting the high retainability of bioactive concentration of VEGF within the implanted site (Tan et al., 2014).

#### 3.3.1 Hydrogel dressings for controlled release of GFs

Hydrogels are three‐dimensional (3D) prehydrated dressings. Because of their expandable, highly crosslinked 3D polymeric network, hydrogels are able to absorb and retain wound exudates, and simultaneously allow water vapor and oxygen transmission to the wound. Hydrogels prepared by 3D polymerization of monomers are physically irreversible; however, due to the generation of significant levels of toxic residual monomers during the polymerization and the likeliness of their leakage from the prepared hydrogels, chemical modifications aimed at developing crosslinking‐ready water‐soluble polymers gained broader acceptance. Adoption of the latter avoided hydrogel purification and allowed use of rapid and more inexpensive crosslinking‐sterilization techniques such as UV‐irradiation (Caló & Khutoryanskiy, 2015). Exposure of FGF‐2 containing photo‐crosslinkable chitosan solution to UV reportedly induced the formation of insoluble and flexible hydrogel within 30 s and its subsequent application facilitated wound closure with complete epithelialization by 16 days in diabetic mice (Obara et al., 2003). In another instance, the high‐binding affinity of bFGF to heparin in the native ECM influenced the development of biomimetic hydrogel films composed of crosslinkable derivatives of chondroitin‐6‐sulfate and heparin for the controlled delivery of bFGF into subcutaneous wounds of genetically diabetic mice. The presence of heparin was speculated to have acted synergistically in modulating the release of bFGF, and allowed optimal healing with low (2 μg) or intermediate (10 μg) dose delivery of bFGF (Liu, Cai, Shu, Shelby, & Prestwich, 2007). Furthermore, a study showed that selective desulfation of heparin could modulate the immobilization efficiency and release of VEGF‐A from StarPEG‐heparin composite hydrogels, implying an attractive avenue for the development of dose‐adjustable GFs delivery system containing sulfated glucosaminoglycans. With increasing desulfation, immobilization efficiency of VEGF‐A was decreased while the initial release kinetics was inversely affected. Implantation of the hydrogel with 0.1 μg or 1 μg VEGF‐A per wound revealed significant granulation tissue and neo‐epithelium formation at 10 days postwounding in diabetic mice (Freudenberg et al., 2015). Alternatively, integration of GF‐conjugation strategies has been found to improve the therapeutic approaches using gels for topical application. One study showed that topical application of dextrin‐rhEGF conjugate formulated at concentrations equivalent to the presence of rhEGF at 1 or 10 μg/ml significantly promoted wound closure in diabetic mice as compared to free rhEGF at 10 μg/ml (Hardwicke et al., 2011). Hydrogels (Hajimiri et al., 2016) and gels (Yeboah et al., 2016) were also employed to deliver GF‐conjugate nanoparticles into diabetic wounds. The nanoparticles employed in the studies were formed by either chemically conjugating the GFs with polymers [e.g. rhEGF‐sodium carboxymethyl chitosan (Hajimiri et al., 2016) or by recombinantly fusing them [e.g. KGF (Koria et al., 2011) or stromal‐derived factor‐1 (SDF‐1) (Yeboah et al., 2016)] with elastin‐like peptides (ELP). Clearly, the emphasis of these studies was with the development of these therapeutic nanoparticles, that offered improved resistance to degradation by protease [savinase (Hajimiri et al., 2016) and elastase (Yeboah et al., 2016)] thereby enhancing the therapeutic effect *in vivo*.

## 4 GENE‐MEDIATED THERAPEUTIC DELIVERY

In principle, gene‐mediated therapeutic delivery involves the localized transfection of therapeutic transgene or complementary DNA (cDNA) into the cells, which then gets transcribed into messenger ribonucleic acid and their translation into the encoded protein *in situ* (Raftery et al., 2016)*.* Initially, the concept of gene therapy was applied to permanent correction of genetic disorders, whose curative effect was assessed by long‐term transgene expression. Conversely, transient gene therapy is of particular interest for local disorders since a transient increase in strategic transgene expression is required until complete tissue repair is achieved (Branski, Gauglitz, Herndon, & Jeschke, 2009). The concept has been put forward for therapeutic investigations in degenerative disorders as it may possibly minimize systemic effects by sustaining high GF concentrations at the targeted site, increase the production of more precisely modified biologically active structure with more recognizable ligands than recombinant proteins. Also, the relatively stable nature with long shelf life of cDNA, ease of large scale production and low‐cost favours the rationale for gene delivery (Southwood, Frisbie, Kawcak, & McIlwraith, 2004). To this end, modulating the degree of gene expression using nonintegrating expression vectors that could avoid undesirable effects arising from host genome integration remains the holy‐grail of regenerative gene medicine (Colosimo, 2000). Typical design strategy is aimed at equipping such vectors for localizing and maintaining extrachromosomally within the host's cell (Jackson, Juranek, & Lipps, 2006). This section will cover the trends in the development of nonintegrating expression vectors and assessment of their transfection efficiency for transient gene therapy in diabetic wound healing. Of particular relevance to wound healing, skin is an amenable organ for genetic manipulations and renders an easy *in vivo* approach as well as follow‐up of therapeutic effects. The high turnover of the epidermis and the fact that a multitude of cytokines and GFs crucial to the regeneration process undergo short‐term up‐ and downregulation make it an ideal target tissue for gene therapy (Bleiziffer, Eriksson, Yao, Horch, & Kneser, 2007).

### 4.1 Delivering plasmid DNAs in diabetic wounds

Naked plasmids represent the simplest form of nonintegrating expression vector. It has a very large DNA packaging capacity and can accommodate large segments of genomic DNA. Regrettably, the large size (1–200 kb) and the presence of phosphodiester backbone offers an overall anionic charge making it difficult for cellular internalization (Raftery et al., 2016). Furthermore, susceptibility of plasmid DNA (pDNA) to rapid clearance by system macrophages and nuclease degradation contributes largely to its low transfection. Therefore, delivery of pDNA into the tissues or cells are assisted with the application of physical methods such as electroporation, sonoporation, hydroporation, laser irradiation, particle bombardment (gene gun) or magnetofection, whose principles are based on improving the kinetics of plasmid transfer via temporal permealization of cell membrane upon application (Mehier‐Humbert & Guy, 2005; Wells, 2004). Early studies on diabetic wounds focussed on forcing the pDNAs into the cells by intradermal injections (Byrnes et al., 2001; Chesnoy, Lee, & Huang, 2003), which later performed in conjunction or adjuvant with electroporation, higher pDNA uptake was achievable (Lee, Chesnoy, & Huang, 2004; Liu et al., 2008; Marti et al., 2004). The relative ease and straightforwardness in applicability, noninvasiveness, ability to target large cell populations and stimulate epithelial cells proliferation (Lee et al., 2004) have perhaps favoured greater use of electroporation as a physical method for pDNA delivery into diabetic wounds. Nevertheless, these techniques require constant optimization of physical parameters (field strength, field distribution and exposure time) depending upon the sensitivity of the tissue. For instance, in diabetic skin, electroporation with low applied voltage of 100 V/cm with 20 ms pulse interval was optimum to avoid tissue damage and at the same time achieve 10‐fold higher transfection than that of normal skin under the same conditions (Lee et al., 2004). Recently, ultrasound micro‐bubbling agents (SonoVue™) assisted sonoporation was used to enhance the delivery of VEGF^165^ encoded minicircles (small modified plasmids) into the wounds of diabetic mice. Application of the ultrasound at a frequency of 1.0 MHz with an exposure intensity and duty cycle at 2.0 W/cm^2^ and 20% respectively, for 30 s was sufficient to maintain higher transfection than pDNAs alone, consequently higher perfusion and wound closure percentage in diabetic mice (Yoon et al., 2009). Principally, the increase in transfection with these methods could be related to higher cytosolic transport of pDNAs, facilitating greater pDNA accumulation in the vicinity of the nucleus. In general, it might serve as a fundamental that applications of any mechanical stimulus be performed with minimized levels of operating parameters as diabetic skins are characterized with reduced dermal thickness and hence, highly susceptible to damage (Petrofsky, Prowse, & Lohman, 2008). This feature of diabetic skin could also account for the deteriorating use of physical methods for pDNA delivery. To further substantiate nuclear transduction, attention has been drawn to the development of carriers that can either deliver the genes directly (viral vectors) or the plasmids encoding the gene of interest (chemical vectors).

### 4.2 Transfection of therapeutic genes with nonintegrating viral vectors

The intrinsic ability of viruses to integrate into host genome and advancements in the design of integration‐ and replication‐defective viruses offer significant advantages in the delivery of therapeutic genes to mammalian cells. Notably, viruses capable of efficiently transfecting both dividing and nondividing cells have been the most explored for diabetic wound healing. These viruses include adenovirus (AV), lentivirus (LV) and adeno‐associated virus (AAV). AV, due to its large genome size packaging capacity (up to 30 kbp) and more importantly, the nonintegrating nature (Boeckle & Wagner, 2006; Zhang & Godbey, 2006) makes it very desirable for use as vector that allows transient expression of the therapeutic gene. One of the early studies reported that transfection of VEGF^165^ gene with a replication‐deficient AV induced earlier wound healing response in diabetic mice leading to an apparent difference in wound closure at day 3 when compared to either untransfected diabetic (control) or nondiabetic mice. Additionally, the transfection of VEGF gene promoted angiogenesis and granulation tissue formation thereby facilitating the recovery of wound at a similar rate as that of nondiabetic mice (Romano Di Peppe et al., 2002). Later in 2009, it was reported that AV mediated VEGF delivery increased keratinocyte migration and collagen deposition, contributing to re‐epithelialization and thicker granulation tissue formation, respectively. The collagen fibres within the neogranulation tissue were found to be long and arranged in an organized manner, the credit for which, was attributed to the presence of AV (Brem et al., 2009). Similar formation of aligned collagen bundles was also observed upon transfecting PDGF gene with LV (Lee et al., 2005) but surprisingly, PDGF gene transferred adenovirally was found to influence random deposition of short collagen fibres (Keswani et al., 2004). Of note, deposition of aligned collagen fibres is a prerequisite for accelerated formation of mature granulation tissue.

The rapid transfection with viral vectors and early induction of healing response suggest that transfection at early injury phase can rescue the wound from progression to its chronicity. For instance, immediate exposure of SDF‐1α (a chemokine) encoded LV post‐wounding, yielded early development of granulation tissue with high dermal cellularity and avoided progression to persistent inflammatory phase in diabetic mice. *In vitro* transfection of dermal fibroblasts by the LV yielded a transfection efficiency of 95% in 3 days (Badillo, Chung, Zhang, Zoltick, & Liechty, 2007). Although it is indicated that the modified non‐integrating LV or AAV may also be suitable for application in wounds desiring longer therapeutic effect of the transgene (Shaw & Cornetta, 2014; Zhang & Godbey, 2006), modulating the expression of transgene for a therapeutically significant period of time has remained a challenging task. Such circumstance was reported by a group where they found that even after the recovery of normalized skin (28 days), VEGF transgene expression mediated via AAVs persisted for up to 4 months *in vivo* (Galeano et al., 2003).

Irrespective of the type of vector or duration of transgene expression, transfection of either a single GF (Botusan et al., 2008) or different GF‐isoform (Saaristo et al., 2006) gene activated only the corresponding signalling pathways, which may not be sufficient to promote the multiple phases of wound healing (Gauglitz & Jeschke, 2011). In light of this problem, the use of a single viral vector capable of transfecting multiple genes (polycistronic viruses) may be advantageous. Design strategies for such viruses have been discussed in detail elsewhere (de Felipe, 2002). Sustained transgene expression for a period of 21 days and the superiority over single GF (FGF4) delivery in diabetic wound healing was demonstrated with the simultaneous delivery of VEGF‐A and FGF4 genes via bicistronic AAVs (Jazwa et al., 2010). All the above studies suggest that the transgene expression was confined to the skin, which is a clinically important setting for avoiding systemic effects to the nearby tissues such as muscle. However, their unpredictable nature such as continual expression beyond a desirable timeline (Galeano et al., 2003) and the risk of causing insertional mutagenesis that occurs as a result of activation of cell‐growth regulatory genes within the virus (Shaw & Cornetta, 2014) remain as major limitations with virus based‐gene delivery. Inconsistency with reproducibility of high titres and purity further limits their progression in applications for gene delivery. To date, refining the safety of viral vectors is maintained as top‐priority for therapeutic translation (Sinn, Sauter, & McCray, 2005). The various delivery routes adopted for the delivery of viral vectors are presented in Table 1.

**Table 1:** Gene delivery vectors applied for the treatment of diabetic wounds

Mode of administration	Therapeutics carrier (applied doses)	Diabetic animal model	Wound geometry	Response on wound closure	References
Intradermal injection	Plasmid KGF‐1 (100 μg)	Genetically diabetic mice	Circular, 5 mm diameter	Enhanced wound closure at day 9	Byrnes et al., 2001
Intradermal injection	Plasmid TGF –β1 (60 μg)	Genetically diabetic mice	Square, 1 × 1 cm	Complete wound closure by 7 days	Chesnoy et al., 2003
Intradermal injection; Electroporation	Plasmid TGF –β1 (30 μg)	Genetically diabetic mice	Square, 7 × 7 mm	Early induction of closure by day 5	Lee et al., 2004
Intradermal injection; Electroporation	Plasmid KGF‐1 (100 μg)	Genetically diabetic mice	Circular, 5 mm diameter	Enhanced wound closure at day 12	Marti et al., 2004
Intradermal injection; Electroporation	Plasmid HIF‐1α (50 μg)	Genetically diabetic mice	Circular, 5 mm diameter	60% wound closure at 10 days	Liu et al., 2008
Subcutaneous injection; Sonoporation	Minicircle‐VEGF (20 μg)	STZ‐diabetic mice	Circular, 6 mm diameter	Complete wound closure by 12 days	Yoon et al., 2009
Topical application to wound bed	Adenovirus encoding VEGF (10^8^ pfu)	STZ‐diabetic mice	Circular, 3.5 mm diameter	Complete wound closure by 13 days	Romano Di Peppe et al., 2002
Intradermal injection	Adenovirus encoding VEGF (5 × 10^10^, 5 × 10^11^, 1.6 × 10^10^, 1.6 × 10^11^ VP)	Genetically diabetic mice	Circular, 1.4 cm diameter	Complete wound closure by 27 days	Brem et al., 2009
Intralesional injection	Adenovirus encoding PDGF (10^8^ pfu)	Genetically diabetic mice	Circular, 8 mm diameter	Residual epithelial gap of 3 mm at day 7	Keswani et al., 2004
Intradermal injection	Adenovirus encoding VEGF‐C (5 × 10^8^ pfu)	Genetically diabetic mice	Circular, 3–5 mm diameter	Complete wound closure by 21 days	Saaristo et al., 2006
Intradermal injection	Adenovirus encoding HIF (10^9^ pfu)	Genetically diabetic mice	Circular, 6 mm diameter	Not defined	Botusan et al., 2008
Injected into base and wound margin	Lentivirus encoding PDGF (10^6^ transducing units)	Genetically diabetic mice	Square, 2 × 2 cm	No statistically significant residual epithelial gap as compared to untreated groups at day 21	Lee et al., 2005
Intradermal injection	Lentivirus encoding SDF‐1α (10^8^ pfu)	Genetically diabetic mice	Circular, 8 mm diameter	Enhanced wound closure at day 7	Badillo et al., 2007
Intradermal injection	Adeno‐associated virus encoding VEGF (10^11^ VP)	Genetically diabetic mice	Not defined	Complete re‐epithelialization at 28 days	Galeano et al., 2003
Intradermal injection	Bicistronic Adeno‐associated virus encoding VEGF‐A and FGF4 (3 × 10^10^ VP)	Genetically diabetic mice	Circular, 4 mm diameter	Complete wound closure by 17 days	Jazwa et al., 2010
Subcutaneous injection	RGDK‐lipopeptide:rhPDGF‐B lipoplex (50 μg pDNA).	STZ‐diabetic rats	Circular, 2.1 cm radius	Complete wound closure by 12 days	Bhattacharyya et al., 2009
Subcutaneous injection	Minicircle VEGF:Cationic dendrimer polyplex (20 μg pDNA)	STZ‐diabetic mice	Not defined	Complete wound closure by 12 days	Kwon et al., 2012

pfu = particle forming units; VP = viral particles

### 4.3 Therapeutic gene delivery using chemical vectors

The emerging interest in the use of chemical vectors such as the cationic polymers and lipids results from their ability to form electrostatic complexes with anionic biomolecules such as the pDNA (Lv, Zhang, Wang, Cui, & Yan, 2006). The adoption of these chemical vectors could not only avoid the use of potentially immunogenic viruses but also improve the biostability of pDNAs, facilitate cellular uptake and undergo endolysosomal escape (Samal et al., 2012). The transfection systems resulting from the use of cationic polymer or lipids are termed polyplex or lipoplex respectively. The transfection efficiency as well as cytotoxicity for such systems is closely dependent upon the charge ratio between the cationic and anionic species in the polymer/lipid and DNA respectively (Lv et al., 2006). Commonly used cationic polymers for gene delivery include polyethyleneimine (PEI) and poly‐l‐lysine (PLL) which are water‐soluble. However, the lipids, because of their amphiphilic nature have gained considerable interest for topical applicability as it can facilitate easy permeation through the multiple hydrophobic‐hydrophilic domains in the skin (Geusens et al., 2011). A study was reported as early as 1997, where combined topical and subcutaneous administration of acidic FGF cDNA with cationic liposomes on a daily basis resulted in increased wound strength and accelerated wound closure in diabetic mice (Sun et al., 1997). Studies in the recent years demonstrated that the versatility for modifications with chemical vectors offers the advantage to translate into more effective carrier for gene delivery that can allow reduction in administration frequency as well as dosage. For instance, single dose subcutaneous injection of lipoplex formed by complexing integrin receptor ligand (RGDK) conjugated lipopetide with rhPDGF‐B (50 μg pDNA) (Bhattacharyya et al., 2009) or polyplex composed of positively charged arginine grafted dendrimer (Starbust) and minicircle VEGF (20 μg pDNA) (Kwon et al., 2012), was able to induce complete wound closure in diabetic mice by 12 days. The induction of healing with lipoplex was attributed to the high selectivity of the ligand for proangiogenic α5β1 receptors on fibroblasts (Bhattacharyya et al., 2009) while with polyplex, it was due to the transfection of rapidly proliferating basal cells (Kwon et al., 2012). Surprisingly, despite these evidences for success, a lack of consistent *in vivo* gene delivery studies using chemical vectors is notable.

### 4.4 Gene‐eluting biomaterial constructs

Development of biomaterial constructs capable of delivering therapeutic genes, as an approach for regenerative therapy is still in its early phase and its application *in vivo*, particularly in diabetic wound healing has been limited to date. The process of developing gene‐eluting biomaterial scaffolds shares similar design principles as that of GF‐loaded scaffolds but with an emphasis directed to improving vector stability and promoting and/or controlling cell‐vector interactions to modulate the location and duration of transgene expression (Seidlits, Gower, Shepard, & Shea, 2013).

In one of the early attempts, Lee, Li, and Huang (2003) developed a purely synthetic (PEG‐PLGA‐PEG) thermosensitive hydrogel capable of *in situ* gelation upon local application to the wound and also controlling the release of encapsulated plasmid (TGF‐β1). The application of the hydrogel elicited significant acceleration of re‐epithelialization at early healing stages (days 1–5) (Lee et al., 2003). This study is in particular, representative of the customizable nature of hydrogels (stimuli sensitive) for spanning the gap between conventional gel‐based systems and solid scaffolds for gene delivery to open wounds. More recent studies have utilised polyplexes, with a focus on modulating their release for efficient gene transfection. In a case using VEGF/PEI polyplex loaded hyaluronic acid hydrogel, the porosity of the hydrogel was considered a fundamental parameter for rapid cellular infiltration, and provide large contact area for maximum encounter of the released polyplexes with the infiltrating cells. However, due to the electrostatic interaction between positively charged polyplexes and anionic hyaluronic acid matrix, the release of polyplex was believed to be very slow to yield high levels of transfected infiltrated cells. Hence the contribution on wound closure as a result of angiogenesis by transfected cells was considered to be insignificant (Tokatlian, Cam, & Segura, 2015). Nevertheless, the fact that the positively charged polyplex interacts electrostatically within the anionic hyaluronic acid hydrogel matrix serves as a basis for the development of more controllable polyplex delivery systems. Yang et al. (2012) showed that the release of polyplex (plasmid bFGF/PEI) encapsulated within the core of core‐sheath emulsion electrospun PELA fibres could be modulated by changing the molecular weights and contents of PEG within the copolymer matrix. Interestingly, their *in vitro* release and transfection period (28 days) was in accordance with the duration of wound recovery *in vivo* (Yang et al., 2012). Surface conjugation of fixed amount of linear PEIs onto nanofibres via matrix metalloproteinase‐responsive linkers offers another strategic approach for developing a polyplex releasing system. Kim and Yoo (2013) found that this strategy allowed tunability in the amount of pDNA to be incorporated in the linear PEI‐immobilized nanofibres and their release as polyplexes following enzymatic cleavage of the linkers resulting in cellular transfection.

In diabetic wounds, issues concerning the low cell availability at the wound edges and their diminished migration (Brem & Tomic‐Canic, 2007; Lerman, Galiano, Armour, Levine, & Gurtner, 2003; Xuan et al., 2014) at the wound site may add to the difficulties in modulating the transfection efficiency *in vivo* with gene‐eluting biomaterial systems. Furthermore, the condition of constant glycation activities in diabetes could present an additional barrier to delivering the desired therapeutic effect. A recent study by Thiersch et al. (2013) found that release of nanocondensates formed by combining PLL‐grafted‐PEG and HIF‐1α plasmid from fibrin hydrogel significantly induced HIF‐1 downstream target VEGF gene expression in healthy animals, but failed to imitate the effect in diabetic animals. The authors implicated that the methylglyoxylation of its transcriptional cofactor p300 inhibited the corresponding downstream signalling cascades (Thiersch et al., 2013).

**Figure 4:**
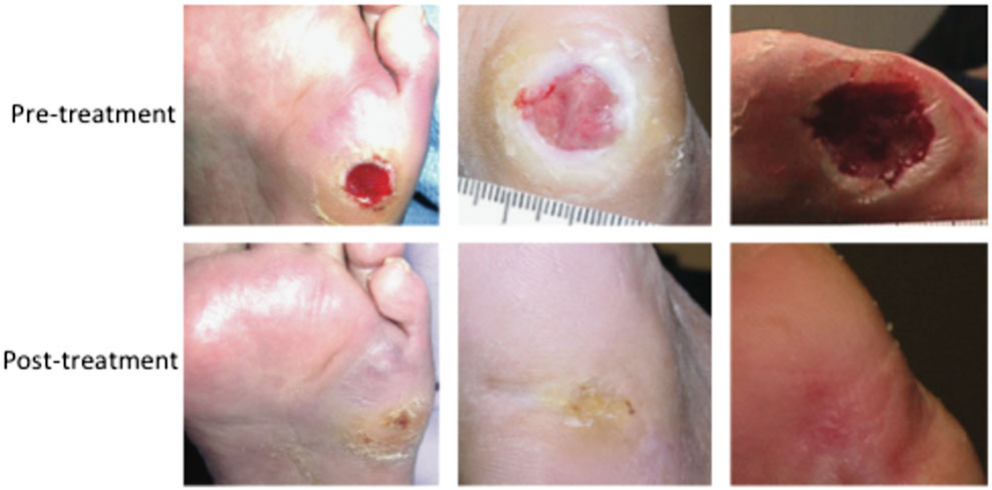
Representative images of healing of foot ulcers following the treatment with GAM501 in phase 1/2 clinical trial (Mulder et al., 2009)

To date, the only clinical trial performed with such gene‐eluting biomaterial systems has been with PDGF‐B encoded replication‐deficient AV formulated within a collagen gel, also termed gene‐activated matrix 501 (GAM501). Patients with ulcer area 1–10 cm^2^ were recruited for the study. Assessment of clinical safety and efficacy conducted in phases 1A and 1B trial found that, a single topical application of GAM501 at 1 × 10^10^, 3 × 10^10^ or 1 × 10^11^ viral particles (VP)/cm^2^ wound bed exhibited similar biologic response as that of multiple applications (up to four topical applications) at single dose level of 3 × 10^10^ VP/cm^2^ at 1‐week intervals (Mulder et al., 2009). One year later, clinical comparison between GAM501, formulated collagen gel and standard of care identified that single application of either GAM501 or formulated collagen gel had a significant healing effect within the first 2 weeks than multiple weekly standard of care interventions (Blume et al., 2011). Figure 4 represents enhanced healing of foot ulcers following the treatment with GAM501 in a phase 1/2 trial.

## 5 STATE OF THE ART AND FUTURE REVIEWS

Having recognized the difficulty imposed upon by wound environment on the bioactivity of localized therapeutics, adoption of an interdisciplinary approach for improving the therapeutics delivery is highly anticipated as a promising solution. As such, our review aimed to provide researchers with information on the various effects corresponding to their rationale on the choice of GF (or gene) or combination of GFs followed by strategic optimization of delivery strategies adopted (injection or topical) for healing of diabetic wounds. Gene delivery has the advantage that it can sustain the production of protein of interest *in situ*; however, the lack of clinically approved chemical vectors and the concerns associated with potentially immunogenic viruses still maintains as major limitations. Additionally, the choice of administrative route could have a significant effect on patient compliance. As is evident from Tables 1 and 2, while gene delivery has been predominantly performed via injection of viral particles encoding for the therapeutic gene, most biomaterial systems have been applied topically, which is relatively less invasive than the former approach. The application of implantable biomaterials not only favours the sustained release of localized concentration of bioactive GFs in the wound site but also offers the advantage of acting as an occlusive without necessitating frequent renewals following implantation. Gene‐eluting biomaterial systems also hold potential for the repair of diabetic wounds, although modulating the cell–vector interaction remains critical for successful induction of the therapeutic effect. In particular relevance to diabetic wound healing, *in vitro* studies relating to gene transfer or angiogenic effect of a particular GF should be performed with cells harvested from diabetic patients, and the succeeding result could be applied for development of more advanced tissue‐engineered wound dressings. Since diabetes is a metabolic disorder, additional strategies targeted to normalizing blood glucose level should also be considered for exerting maximum effect of the delivered therapeutics.

**Table 2:** Biomaterial systems applied for the delivery of growth factors and genes in diabetic wounds

Particulate systems for the sustained release of growth factors into diabetic wounds					
Delivery route	Particulate system (loading amount)	Diabetic animal model	Wound geometry	Response on wound closure	Reference
Topical spraying to wound bed	rhEGF loaded PLGA nanoparticles (50 μl of rhEGF at 0.1 μg/μl)	STZ‐diabetic rats	Circular, 1.8 cm diameter	Complete wound closure by 21 days	Chu et al., 2010
Intradermal injection	VEGF loaded PLGA nanoparticles (nps; 1.26 μg of VEGF/mg of PLGA nps)	Genetically diabetic mice	Circular, 6 mm diameter	Complete wound closure by 19 days	Chereddy et al., 2015
Intralesional injection	rhEGF loaded PLGA‐Alginate microspheres (1% *w*/w)	STZ‐diabetic rats	Circular, 1 cm diameter	90% wound closure at 11 days	Gainza et al., 2013
Topical application to wound bed	rhEGF loaded lipid nanocarriers (1% *w*/w)	Genetically diabetic mice	Circular, 8 mm diameter	95% wound closure at 15 days	Gainza et al., 2014

Locally implantable biomaterial systems for growth factors delivery to diabetic wound bed					
Type of implant	Type of formulation (loading amount)	Diabetic animal model	Wound geometry	Response on wound closure	Reference
Non‐woven mesh (Electrospun)	rhPDGF/PLGA (0.005 g/0.28 g)	STZ‐diabetic rats	Circular, 8 mm diameter	Complete wound closure by 14 days	Lee et al., 2015
	rhEGF/PCL, PCL‐PEG (0.00072 nmol/mg)	STZ‐diabetic mice	Circular, 8 mm diameter	Accelerated wound closure at 7 days	Choi et al., 2008
	bFGF/PELA (0.079 ± 0.015% *w*/w)	STZ‐ diabetic rats	Circular, 250 mm^2^	Complete wound closure by 3 weeks	Yang et al., 2011
	bFGF, rhEGF/PCL, PCL‐PEG (0.98 μg of bFGF and 9.4 μg of rhEGF per device)	STZ‐ diabetic rats	Circular, 8 mm diameter	Accelerated wound closure at 7 days	Choi et al., 2011
	VEGF, bFGF, EGF, PDGF/collagen, hyaluronic acid, gelatine nps (5 μl of VEGF or PDGF at 0.2 μg/ml per 10 mg of gelatine nps)	STZ‐ diabetic rats	Circular, 15 mm diameter	Complete wound closure by 4 weeks	Lai et al., 2014
Woven nylon mesh	VEGF, PDGF/poly(β‐amino esters), poly(acrylic acid), Heparan sulfate (40 tetralayer repeats of PDGF or VEGF per section)	Genetically diabetic mice	Circular, 6 mm diameter	Accelerated wound closure at 14 days	Almquist et al., 2015
Porous scaffolds	bFGF/collagen, gelatine (7–14 μg/cm^2^)	Genetically diabetic mice	Circular, 8 mm diameter	Not defined	Kanda et al., 2014
	EGF/collagen, hyaluronic acid (2 μg/cm^2^)	Genetically diabetic mice	1.5 × 2 cm	Not defined	Kondo et al., 2012
	aFGF/collagen, chitosan (4 μg/2.5 cm^3^)	STZ‐ diabetic rats	Circular, 1.8 cm diameter	Complete healing at 14 days	Wang et al., 2008
	bFGF/collagen, PGA (100μl at 100 μg/ml)	Genetically diabetic mice	Circular, 6 mm diameter	Not defined	Nagato et al., 2006
	VEGF/bFGF loaded PLGA nps, fibrin (20 mg of nps containing 200ng of VEGF or bFGF)	Genetically diabetic mice	Circular, 8 mm diameter	85% wound closure at 15 days	Losi et al., 2013
	rhEGF/PU (8.4μg/cm^2^)	STZ‐ diabetic rats	Square, 2 × 2 cm	97% wound closure at 21 days	Pyun do et al., 2015
Hydrogels	FGF‐2/Chitosan (250 ng/50 μl)	Genetically diabetic mice	Circular, 100 mm^2^	80% wound closure by 12 days	Obara et al., 2003
	bFGF/Chondroitin‐6‐sulfate, Heparin (20 μg/film)	Genetically diabetic mice	Circular, 1.6 cm diameter	89% wound closure by 2 weeks (20 μg)	Liu et al., 2007
	VEGF/PEG, Heparin (0.1 or 1 μg/gel)	Genetically diabetic mice	Not defined	Not defined	Freudenberg et al., 2015
	rhEGF‐Sodium carboxymethyl chitosan nps/ PVP (0.25 ml of nps solution/2.5 ml of PVP)	STZ‐diabetic rats	Circular, 2 cm diameter	Negligible wound area at day 15	Hajimiri et al., 2016
**Gene‐eluting biomaterial systems applied for the treatment of diabetic wounds**					
Hydrogels	pDNA TGF‐β1/PEG‐PLGA‐PEG (200 μg/70 μl)	Genetically diabetic mice	Square, 7 × 7 mm	Accelerated wound closure at 5 days	Lee et al., 2003
	pDNAVEGF‐PEI polyplex/Hyaluronic acid (lyophilized polylex containing 250 μg pDNA per 100 μl gel)	Genetically diabetic mice	Circular, 6 mm diameter	Induction of wound closure by day 8–10	Tokatlian et al., 2015
	pDNA HIF‐PLL grafted PEG nanocondensates/Fibrin (nanocondensates carrying 1 μg pDNA per 100 μl gel)	STZ‐diabetic rats	Circular, 10 mm diameter	Not defined	Thiersch et al., 2013
Electrospun mesh	pDNA bFGF‐PEI polyplex/PELA (50 μl of polyplex solution at 1 mg/ml pDNA per 450 mg PELA)	STZ‐diabetic rats	Circular, 250 mm^2^	Complete wound closure by 3 weeks	Yang et al., 2012
	pEGF‐linear PEI/PCL‐PEG (6.8 μg/device)	STZ‐diabetic mice	Circular, 9 mm diameter	Enhanced wound closure at day 7	Kim & Yoo, 2013

## CONFLICT OF INTEREST

The authors declare that no conflict of interest exists.
